# Screening for dysphagia in older people with dementia: Evidence of validity based on internal structure and reliability of the Caregiver Questionnaire – RaDID-QC

**DOI:** 10.1016/j.clinsp.2024.100440

**Published:** 2024-08-09

**Authors:** Grazielle Duarte de Oliveira, Laélia Cristina Caseiro Vicente, Aline Mansueto Mourão, Sayuri Hiasmym Guimarães Pereira dos Santos, Uriel Moreira Silva, Amélia Augusta de Lima Friche, Maria Aparecida Camargos Bicalho

**Affiliations:** a Sciences Applied to Adult Health at the Universidade Federal de Minas Gerais, Belo Horizonte, MG, Brazil; b Department of Speech-Language-Hearing Sciences of the Faculdade de Medicina, Universidade Federal de Minas Gerais, Belo Horizonte, MG, Brazil; c Faculdade de Medicina, Universidade Federal de Minas Gerais, Belo Horizonte, MG, Brazil; d Department of Statistics (DEST), of ICEx at the Universidade Federal de Minas Gerais, Belo Horizonte, MG, Brasil; e Department of Medicine of the Faculdade de Medicina, Universidade Federal de Minas Gerais, Belo Horizonte, MG, Brazil; f Member of the National Institute for Responsible Neurotechnology (INCT Neurotec-R). Geriatrician at the Jenny de Andrade Faria Reference Center for the elderly – University Hospital of the Universidade Federal de Minas Gerais, Brazil

**Keywords:** Dementia, Swallowing Disorders, Caregivers, Surveys and questionnaires, Older adults

## Abstract

**Objective:**

To identify internal structure validity evidence of a dysphagia screening questionnaire for caregivers of older adults with Alzheimer's disease dementia and/or vascular dementia.

**Methods:**

The 24-question Dysphagia Screening in Older Adults with Dementia – Caregiver Questionnaire (RaDID-QC) was administered by interviewing 170 caregivers of older people with dementia, selected by convenience at the Outpatient Reference Center for Older People. Exploratory Factor Analysis (EFA) was used to assess the internal structure validity of the questionnaire, and Cronbach's alpha was used to analyze reliability. Questions with factor loadings lower than 0.45 in magnitude were removed from the final questionnaire. Multivariate multiple linear regression was used to assess the percentage of variance explained by the remaining questions.

**Results:**

Kayser-Meyer-Olkin (KMO) and Bartlett's tests suggested that the questionnaire was adequate for EFA. Principal Component Analysis (PCA) suggested that 12 components captured at least 75 % of the total variance. The corresponding 12-factor EFA model showed a statistically significant fit, and 15 out of the 24 questions had factor loadings greater than 0.45. Cronbach's alpha was 0.74 for the 15 questions, which explained 71 % of the total variance in the complete dataset. The questionnaire has adequate internal structure validity and good reliability. Based on EFA, RaDID-QC decreased from 24 to 15 questions. Other internal validity and reliability parameters will be obtained by administering the questionnaire to larger target populations.

**Conclusion:**

The RaDID-QC applied to caregivers of older adults with dementia due to Alzheimer's disease and/or vascular dementia produced valid and reliable responses to screen dysphagia signs and symptoms.

## Highlights

• RaDID-QC was developed to screen dysphagia signs and symptoms.
• RaDID-QC is meant to be applied to caregivers of older people with dementia.
• RaDID-QC is a simple, concise, easy-to-apply, quick, and reliable questionnaire.

## Introduction

Alzheimer's Disease (AD) is a neurodegenerative disease that affects 50 % to 60 % of older people with dementia. Vascular Dementia (VD), the second most common cause of dementia, accounts for approximately 17 % to 30 % of all cases.^1^

The various causes of dementia impair different brain regions and cognitive functions, resulting in varied forms of Oropharyngeal Dysphagia (OD), a common clinical manifestation in this population.^2^ In general, AD patients predominantly have sensory dysfunctions, while individuals with VD have motor swallowing impairments, characterized by difficulties in food bolus formation and propulsion through the pharynx and a greater degree of silent aspirations.^2^

Swallowing impairment can affect 80 % to 93 % of individuals^3^, ^4^, ^5^, ^6^, ^7^, ^8^, ^9^ with Alzheimer's Disease Dementia (ADD) in the moderate and advanced stages when cognitive and motor functions are severely impaired.^10^ In mild ADD, 30.8 % to 45.5 % of patients may experience OD.^7^^,^^10^ However, the most frequent changes are subtle, found through videofluoroscopic swallowing studies.^5^ Patients and caregivers often do not recognize dysphagia, which contributes to its underdiagnosis,^10^ preventing or delaying the implementation of rehabilitative measures aimed at reducing complications.

Screening questionnaires are simple, low-cost, and easy to apply. Although there are validated questionnaires to identify dysphagia in older adults with preserved cognition,^11^, ^12^, ^13^, ^14^, ^15^ the literature has no dysphagia screening instruments for those with dementia.

Older people with dementia may be unable to recognize food visually and have tactile and oral agnosia, swallowing apraxia, and difficulties in providing reliable information,^9^^,^^16^ whereas the caregiver is usually able to provide them reliably.^17^

Currently, there are validated screening instruments for identifying dysphagia in cognitively unimpaired older adults.^12^^,^^14^^,^^15^^,^^18^, ^19^, ^20^ In addition, there is a questionnaire constructed to investigate caregiver burden related to dysphagia.^21^ Nevertheless, to the best of our knowledge, to date, no dysphagia screening questionnaires applied to caregivers of older adults with dementia have been described in the literature. This type of instrument could improve the recognition of swallowing disorders in older adults with dementia since this population is not able to recognize this kind of dysfunction.

To fill the gap in the literature, the authors developed the “Dysphagia Screening in Older People with Dementia – Caregiver Questionnaire” (RaDID-QC, in Portuguese) to identify DO in older people with ADD and/or mild, moderate, or advanced DV by interviewing their caregivers. RaDID-QC has presented evidence of validity based on content and response processes in a previous stage.

This study aimed to identify the validity of the internal structure and internal consistency of RaDID-QC, and evaluate the possibility of reducing the number of its questions.

## Materials and methods

The authors followed the STARD guidelines for reporting the results of this study.^22^

This is a cross-sectional, observational, validation study, whose procedures to validate the instrument's internal structure and reliability followed the Standards for Educational and Psychological Testing guidelines.^23^

The study was approved by the Research Ethics Committee under evaluation report number 4.952.238. All participants received instructions and signed an informed consent form.

The older adults and their caregivers were selected by convenience. The patients were outpatients at the Jenny de Andrade Faria Institute – a Reference Center for Older People at the University Hospital of the Universidade Federal de Minas Gerais (UFMG). The study was carried out from 2019 to 2023.

Older adults were, initially, evaluated by a geriatrician. The diagnosis of ADD was based on the McKhann criteria,^24^ and that of VD was based on DSM-5 criteria (2014).^25^^,^^26^ The severity of dementia was classified according to the Clinical Dementia Rating (CDR).^27^^,^^28^ The patients’ sociodemographic (sex, age, and education) and clinical data were collected from medical records and confirmed with their caregivers.

The caregivers’ sociodemographic data (sex, age, education, and socioeconomic conditions [according to the Brazilian Economic Classification Criteria – CCEB])^29^ were obtained through interviews. Caregivers underwent cognitive screening with the Mini-Mental State Examination (MMSE).^30^

The patients/caregivers met the following inclusion criteria: the older adults had to be 60 years or older and have a diagnosis of mild, moderate, or advanced ADD and/or VD. Caregivers had to be 18 years or older, provide formal or informal assistance to the older adult, agree to participate, and sign an informed consent form.

The authors excluded older people with a clinical diagnosis of stroke or other neurological diseases and those previously evaluated by a speech-language-hearing pathologist (to avoid the influence of information on dysphagia) from the sample of the study. The authors also excluded caregivers who had been previously instructed on dysphagia, who were unable to understand the procedures or respond to the questionnaire due to hearing loss, or whose MMSE results^30^ were below the cutoff for their education level.^31^^,^^32^

After selecting the patients/caregivers, a speech-language-hearing pathologist interviewed the caregivers individually with the RaDID-QC. Each Question (Q) had five answer options: “never”, “few times”, “sometimes”, “most of the time” and “every time”, which were answered considering the frequency of each event in the last month. Caregivers were instructed to answer the questions based on the following guidelines: NEVER means that the requested event not at any time; FEW TIMES, when the event has happened rarely; SOMETIMES, when the event has happened occasionally; MOST OF THE TIME, when the event happened many times; EVERY TIME, when the event has happened all the time.

The sample size was calculated considering at least five times more observations than the number of questions, which resulted in a minimum of 120 individuals.^33^

Regarding the internal structure validity of the scale, a preliminary Principal Component Analysis (PCA) was conducted to define the number of factors to be applied for the Exploratory Factor Analysis (EFA), undertaken to evaluate the validity of the internal structure of RaDID-QC regarding the distribution of questions. The adequacy of EFA to RaDID-QC was analyzed with the Kayser-Meyer-Olkin (KMO) and Bartlett Sphericity (BTS) tests. The internal reliability of the complete scale was assessed with Cronbach's alpha.

The authors produced a shortened version of RaDID-QC by retaining only questions with factor loadings at least 0.45 in magnitude. Additionally, the authors used a multivariate multiple linear regression to assess the variability from the full RaDID-QC retained in the shortened version. Finally, the reliability of the shortened version was reassessed with Cronbach's alpha.

All analyses were performed in the R software environment, version 4.3.1.^34^

## Results

In total, 170 patients/caregivers participated in the study. The older adults were 60 to 97 years old (mean of 80 years, SD±7.07), most of whom were women (68.2 %) who had attended school for 1 to 4 years (53.5 %). AD was the main cause of dementia (94 %) (Table 1).

**Table 1:** Older adults’ sociodemographic and clinical characteristics and caregivers’ sociodemographic characteristics.

Older people		N	%
Sex	Males	54	31.8
	Females	116	68.2
Age	60 to 69 years	17	10.0
	70 to 79 years	64	38.0
	+80 years	89	52.0
Education level	Illiterate	52	30.5
	Up to 4 years	91	53.5
	Up to 8 years	7	4.0
	Up to 11 years	15	9.0
	More than 11 years	5	3.0
Type of dementia	Alzheimer	159	94.0
	Vascular	11	6.0
CDR	Mild	62	36.0
	Moderate	64	38.0
	Advanced	44	26.0
**Caregivers**		**n**	**%**
Sex	Males	25	15.0
	Females	145	85.0
Age	24 to 29 years	6	3.5
	30 to 39 years	14	8.0
	40 to 49 years	47	28.0
	50 to 59 years	56	33.0
	60 to 69 years	32	19.0
	70 to 79 years	14	8.0
	+80 years	1	0.5
Education level	Illiterate	3	2.0
	Up to 4 years	31	18.0
	Up to 8 years	17	10.0
	More than 11 years	73	43.0
Type of caregiving	Informal	163	96.0
	Formal	7	4.0
Resides with the patient	No	80	47.0
	Yes	90	53.0
Daily workload	Up to 12 h	72	42.0
	More than 12 h	98	58.0
Weekly workload	1 day	8	4.7
	2 days	12	7.0
	3 days	10	5.8
	4 days	9	5.0
	5 days	11	7.0
	6 days	5	2.9
	7 days	115	67.6
CCEB	Class A	2	1.18
	Class B1	17	10.0
	Class B2	38	22.35
	Class C1	50	29.41
	Class C2	44	25.88
	Class D/E	19	11.18

CDR, Clinical Dementia Rating; CCEB, Brazilian Economic Classification Criteria.

Caregivers were 24 to 87 years old (mean of 53 years; SD±12.05 years), 85 % were women, 70 % had attended school for 9 or more years, most of them (96 %) provided informal assistance, 53 % lived with the older adult, 58 % stayed with them 12 or more hours a day, and 68 % stayed with them 7 days a week (Table 1).

RaDID-QC took 10 min at the most to administer.

The descriptive analysis results of the five possible answers for the 22 RaDID-QC questions and the three possible answers for two questions are described in Table 2. The mean answers for almost all questions ranged from never (1) to few times (2), except for Q24, in which never prevailed (1.14).

**Table 2:** Description of the caregivers’ responses to the 24 RaDID-QC questions.

	Caregivers’ responses			
Questions (Q)	Mean	SD	Min.	Max.
1. Have you noticed if the older adult has difficulty recognizing foods?	2.04	1.54	1	5
2. Does the older adult refuse to eat?	1.92	1.22	1	5
3. Have you noticed if the older adult is taking longer than usual to eat their meals?	2.54	1.73	1	5
4. Does the older adult have difficulties eating alone and need help?	1.54	1.23	1	5
5. Does the older adult have difficulties taking food from a spoon/fork or drinking from a cup?	1.45	1.15	1	5
6. Does the older adult need any specific utensil to eat better?	1.17	0.80	1	5
7. Does the older adult put an exaggerated amount of food in their mouth?	1.38	1.05	1	5
8. During meals, does the older adult let food or liquid spill out of their mouth?	1.70	1.22	1	5
9. Do you notice saliva drooling out of the older adult's mouth when they are awake?	1.19	0.75	1	5
10. Does the older adult have difficulties or forget to chew food?	1.54	1.20	1	5
11. Does the older adult forget or take long to swallow saliva, food, or liquids?	1.35	0.93	1	5
12. Do you have to ask the older adult to swallow the food?	1.31	0.87	1	5
13. Does the older adult cough, choke, or clear the throat during meals?	2.00	1.21	1	5
14. Does the older adult cough, choke, or clear the throat after meals?	1.51	1.02	1	5
15. Does the older adult cough, clear the throat, or choke on saliva?	1.59	1.00	1	5
16. Have you noticed if the older adult has to make an effort to swallow?	1.29	0.80	1	5
17. Does the older adult have pain or any discomfort (e.g., breathlessness, tiredness) when they are eating?	1.16	0.54	1	4
18. Does the older adult have food left in their mouth after swallowing?	1.51	1.20	1	5
19. Does the older adult's voice change after swallowing?	1.17	0.66	1	5
20. Have you ever noticed food or liquid coming out the older adult's nose?	1.05	0.27	1	3
21. Does the food the older adult swallowed return after eating (gastroesophageal reflux)	1.41	1.00	1	5
22. Does the older adult have difficulties swallowing pills?	1.65	1.32	1	5
23. Have you noticed any weight loss in the last 3 months due to eating difficulties?	1.48	0.84	1	3
24. Did the older adult have pneumonia within the last year?	1.14	0.36	1	3

Q, Questions; SD, Standard Deviation; min, minimum; max, maximum; Q1 to Q22: 1 = never, 2 = seldom, 3 = sometimes, 4 = usually, 5 = always; Q23 – 1 = no, 2 = I don't know, 3 = yes; Q24 – 1 = never, 2 = once, 3 = two or more times.

### Exploratory factor analysis

RaDID-QC had a KMO of 0.67 and p < 0.001 in BTS.

The PCA suggested that 12 components captured at least 75 % of the total variance; therefore, this was the number of factors chosen for the EFA. Along with the PCA results, the authors also considered the questions' correlation matrix, the corresponding scree plot, and Kayser's rule to decide on the number of factors. Full details are provided in the Supplement.

The 12-factor EFA model fitted across all RaDID-QC questions showed a statistically significant fit. The Chi-Square goodness-of-fit test, of which 12 factors were sufficient to explain the variability in the data, had a p-value of 0.507. Overall, 15 of the 24 questions had factor loadings greater than 0.45, and therefore only these were retained to form the shortened questionnaire. These 15 questions explained 71 % of the total variance in the full RaDID-QC's 24 questions (Table 3).

**Table 3:** Exploratory Factor Analysis (EFA) of the 24 RaDID-QC questions.

Questions (Q)	F1	F2	F3	F4	F5	F6	F7	F8	F9	F10	F11	F12
1. Have you noticed if the older adult has difficulty recognizing foods?	0.39	0.13	0.11	0.06	-0.04	0.03	0.24	0.02	0.30	-0.13	-0.20	0.28
**2. Does the older adult refuse to eat?**	0.03	-0.03	0.04	-0.01	-0.01	-0.09	0.11	0.04	0.07	0.09	**0.55**	0.22
3. Have you noticed if the older adult is taking longer than usual to eat their meals?	0.16	0.04	-0.07	0.16	0.03	0.05	0.26	0.11	-0.04	0.12	0.01	0.33
**4. Does the older adult have difficulties eating alone and need help?**	**0.76**	0.04	0.02	0.07	0.04	-0.08	0.03	0.11	0.04	0.02	-0.14	0.13
**5. Does the older adult have difficulties taking food from a spoon/fork or drinking from a cup?**	**0.93**	0.08	0.05	0.11	0.01	0.10	0.08	0.01	-0.07	0.10	0.07	-0.09
6. Does the older adult need any specific utensil to eat better?	0.13	0.05	0.17	0.03	0.01	-0.11	-0.07	0.06	0.10	0.09	-0.29	0.01
7. Does the older adult put an exaggerated amount of food in their mouth?	0.02	0.23	-0.05	0.28	-0.07	0.10	0.16	0.02	0.26	0.40	-0.23	-0.06
**8. During meals, does the older adult let food or liquid spill out of their mouth?**	0.11	**0.94**	0.03	0.18	0.08	0.04	0.13	0.02	0.00	0.21	0.01	0.09
9. Do you notice saliva drooling out of the older adult's mouth when they are awake?	0.07	0.42	0.32	0.06	0.07	0.03	-0.14	0.02	0.21	-0.02	0.21	-0.16
**10. Does the older adult have difficulties or forget to chew food?**	0.24	0.15	0.05	**0.91**	0.03	0.03	0.10	0.13	0.12	0.18	0.00	0.05
**11. Does the older adult forget or take long to swallow saliva, food, or liquids?**	0.23	0.03	-0.01	0.04	-0.01	0.04	**0.61**	0.05	0.03	0.05	0.11	0.09
**12. Do you have to ask the older adult to swallow the food?**	**0.55**	-0.02	0.05	0.07	0.03	-0.07	0.37	0.13	0.07	0.01	0.12	0.04
**13. Does the older adult cough, choke, or clear the throat during meals?**	0.07	0.10	0.17	0.13	0.11	0.19	0.01	0.06	0.25	**0.64**	0.19	0.09
**14. Does the older adult cough, choke, or clear the throat after meals?**	0.03	0.03	-0.02	0.06	0.06	0.11	0.02	0.02	**0.78**	0.22	0.12	0.05
**15. Does the older adult cough, clear the throat, or choke on saliva?**	0.02	0.20	0.04	0.05	0.08	0.02	0.02	0.07	0.26	0.03	**0.45**	-0.14
**16. Have you noticed if the older adult has to make an effort to swallow?**	-0.02	0.10	**0.50**	0.13	0.19	-0.03	**0.47**	0.09	0.02	0.09	0.18	-0.02
**17. Does the older adult have pain or any discomfort (e.g., breathlessness, tiredness) when they are eating?**	0.05	0.09	0.13	0.01	**0.98**	-0.01	0.02	-0.02	0.06	0.04	0.03	0.07
18. Does the older adult have food left in their mouth after swallowing?	0.36	0.23	0.10	-0.05	-0.05	0.00	0.27	0.15	0.00	0.38	-0.23	0.01
**19. Does the older adult's voice change after swallowing?**	-0.02	0.05	0.07	0.07	0.00	**0.96**	0.03	0.13	0.14	0.17	0.00	0.02
**20. Have you ever noticed food or liquid coming out the older adult's nose?**	0.09	0.04	**0.84**	0.01	0.07	0.08	0.01	-0.01	-0.03	0.07	-0.07	0.03
21. Does the food the older adult swallowed return after eating (gastroesophageal reflux)	-0.10	0.03	0.09	0.04	0.20	0.10	0.12	0.10	0.18	0.17	0.05	-0.38
**22. Does the older adult have difficulties swallowing pills?**	0.22	0.03	0.03	0.08	-0.01	0.14	0.14	**0.95**	0.03	0.07	0.02	0.01
23. Have you noticed any weight loss in the last 3 months due to eating difficulties?	-0.02	0.00	0.04	0.01	0.09	0.03	0.06	0.01	0.08	0.05	0.09	0.40
24. Did the older adult have pneumonia within the last year?	-0.01	0.14	0.13	0.23	0.00	0.13	0.03	-0.13	-0.04	-0.07	0.23	0.07
Cronbach's alpha	0.78											

Q, Questions. Values in bold are factor loads ≥0.45. Questions in bold were selected for the final/short version of the RaDID-QC (Dysphagia Screening in Older People with Dementia). The EFA adequacy test had a p-value of 0.507. F, Factor.

**Chart 1:**
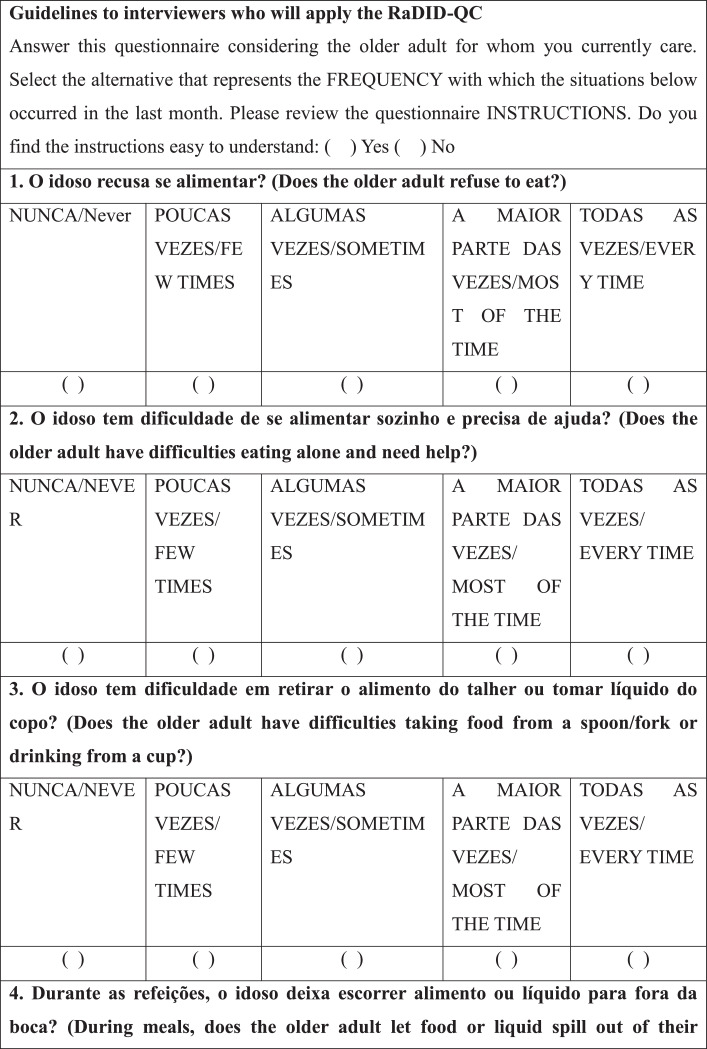

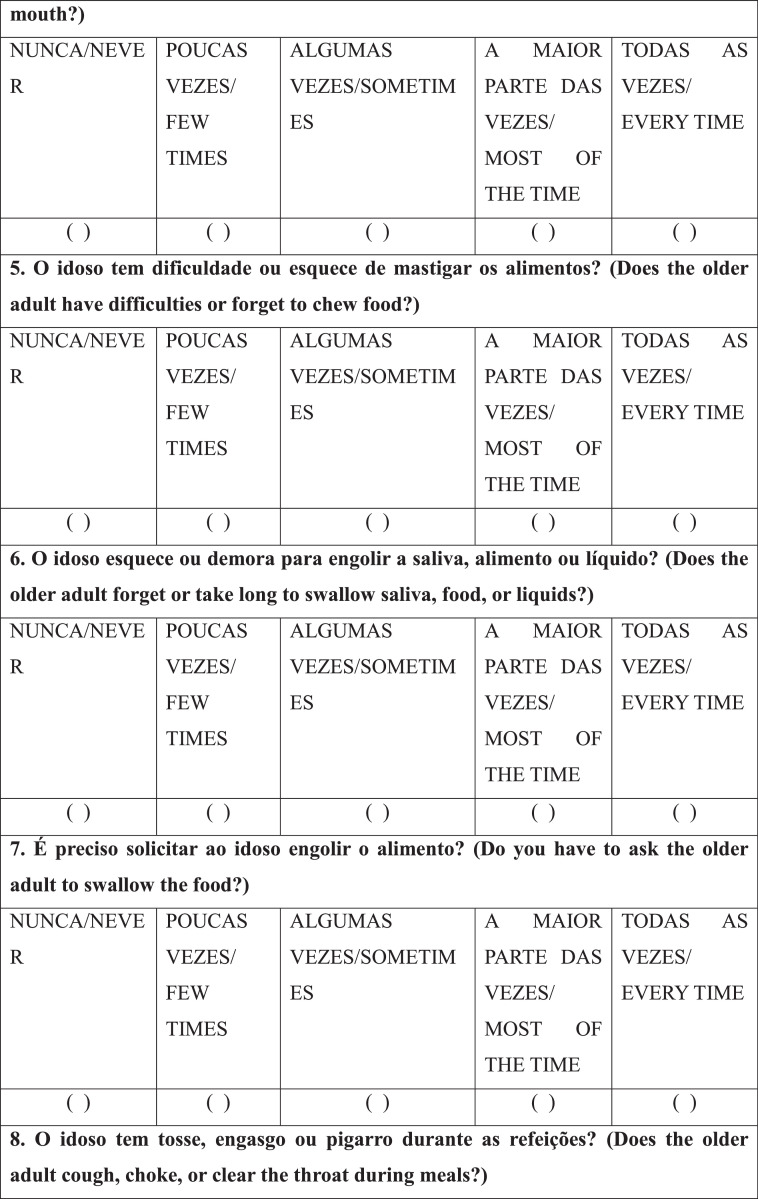

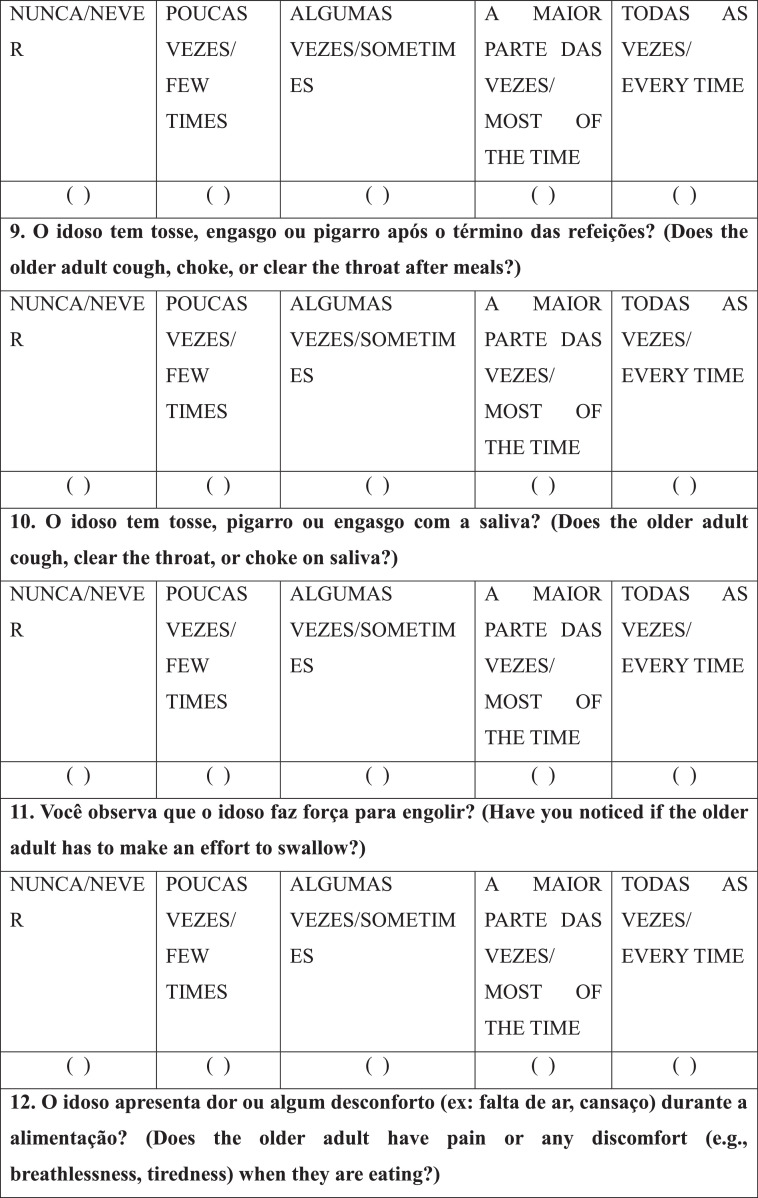

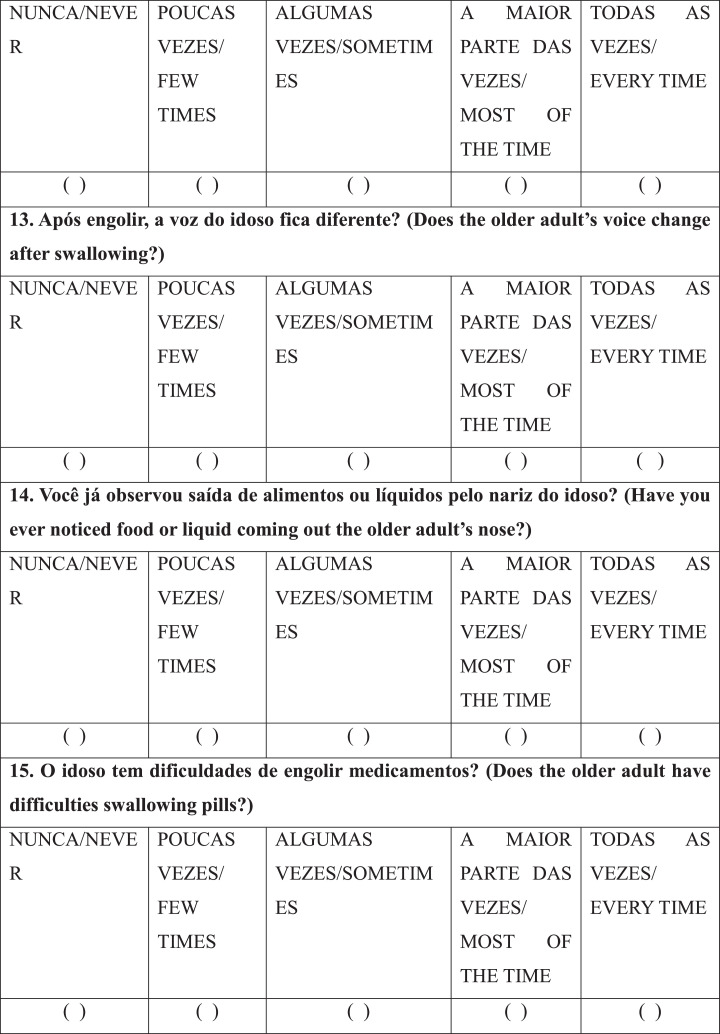
Final version of the Dysphagia Screening in Older People with Dementia – Caregiver Questionnaire (RaDID-QC)a. ^a^The translation of RaDID-QC from Portuguese to English was done for publication purposes without the steps necessary for transcultural translation and adaptation to the English language. Instruções/Instructions: NUNCA/NEVER: significa que no evento não ocorreu em nenhum momento/means that the requested event not at any time; POUCAS VEZES/FEW TIMES: quando o evento ocorreu de forma rara/when the event has happened rarely; ALGUMAS VEZ/SOMETIMES: quando o evento ocorreu ocasionalmente/when the event has happened occasionally; A MAIOR PARTE DAS VEZES/MOST OF THE TIME: quando o evento ocorreu muitas vezes/when the event happened many times; TODAS AS VEZES/EVERY TIME: quando o evento ocorreu todas as vezes/when the event has happened all the time.

Finally, regarding internal reliability, Cronbach's alpha was 0.78 for the full RaDID-QC questionnaire (Table 3) and 0.74 for the shortened questionnaire (Table 4). The shortened RaDID-QC questionnaire can be found in Chart 1.

**Table 4:** Exploratory factor analysis of the 15 RaDID-QC questions.

Questions (Q)	F1	F2	F3	F4	F5	F6	F7	F8	F9	F10	F11	F12
1. Does the older adult refuse to eat?	0.03	-0.03	0.04	-0.01	-0.01	-0.09	0.11	0.04	0.07	0.09	**0.55**	0.22
2. Does the older adult have difficulties eating alone and need help?	**0.76**	0.04	0.02	0.07	0.04	-0.08	0.03	0.11	0.04	0.02	-0.14	0.13
3. Does the older adult have difficulties taking food from a spoon/fork or drinking from a cup?	**0.93**	0.08	0.05	0.11	0.01	0.10	0.08	0.01	-0.07	0.10	0.07	-0.09
4. During meals, does the older adult let food or liquid spill out of their mouth?	0.11	**0.94**	0.03	0.18	0.08	0.04	0.13	0.02	0.00	0.21	0.01	0.09
5. Does the older adult have difficulties or forget to chew food?	0.24	0.15	0.05	**0.91**	0.03	0.03	0.10	0.13	0.12	0.18	0.00	0.05
6. Does the older adult forget or take long to swallow saliva, food, or liquids?	0.23	0.03	-0.01	0.04	-0.01	0.04	**0.61**	0.05	0.03	0.05	0.11	0.09
7. Do you have to ask the older adult to swallow the food?	**0.55**	-0.02	0.05	0.07	0.03	-0.07	0.37	0.13	0.07	0.01	0.12	0.04
8. Does the older adult cough, choke, or clear the throat during meals?	0.07	0.10	0.17	0.13	0.11	0.19	0.01	0.06	0.25	**0.64**	0.19	0.09
9. Does the older adult cough, choke, or clear the throat after meals?	0.03	0.03	-0.02	0.06	0.06	0.11	0.02	0.02	**0.78**	0.22	0.12	0.05
10. Does the older adult cough, clear the throat, or choke on saliva?	0.02	0.20	0.04	0.05	0.08	0.02	0.02	0.07	0.26	0.03	**0.45**	-0.14
11. Have you noticed if the older adult has to make an effort to swallow?	-0.02	0.10	**0.50**	0.13	0.19	-0.03	**0.47**	0.09	0.02	0.09	0.18	-0.02
12. Does the older adult have pain or any discomfort (e.g., breathlessness, tiredness) when they are eating?	0.05	0.09	0.13	0.01	**0.98**	-0.01	0.02	-0.02	0.06	0.04	0.03	0.07
13 Does the older adult's voice change after swallowing?	-0.02	0.05	0.07	0.07	0.00	**0.96**	0.03	0.13	0.14	0.17	0.00	0.02
14. Have you ever noticed food or liquid coming out the older adult's nose?	0.09	0.04	**0.84**	0.01	0.07	0.08	0.01	-0.01	-0.03	0.07	-0.07	0.03
15. Does the older adult have difficulties swallowing pills?	0.22	0.03	0.03	0.08	-0.01	0.14	0.14	**0.95**	0.03	0.07	0.02	0.01
Cronbach's alpha			0.74									

Q, Questions; Values in bold are factor loads ≥0.45; RaDID-QC, Dysphagia Screening in Older People with Dementia; F, Factor.

## Discussion

The RaDID-QC aims to screen dysphagia signs and symptoms in older people with dementia to avoid complications related to swallowing safety and efficiency. The dissemination of RaDID-QC provides better care management and helps avoid complications, promoting quality of life and health for older adults with dementia.

No similar instruments were found in the literature analyzed, such as those administered to caregivers of older people with dementia to screen OD. The lack of instruments for this purpose restricts this population's access to instructions and information and contributes to the underdiagnosis of dysphagia.

Moreover, a systematic review^35^ on the prevalence of OD analyzed three studies with self-reported screening questionnaires^11^, ^12^, ^13^ and identified low methodological quality and flaws in the description of psychometric properties. Two studies had flaws in the planning and execution of factor analysis,^11^^,^^12^ and the third one^13^ had no factor rotation.

The Screening of Oropharyngeal Dysphagia in Older Adults (RaDI) – a questionnaire with perspectives similar to those of the RaDID-QC – was developed and validated for older people with preserved cognition.^14^ Sheikhany and collaborators developed an instrument to screen dysphagia and eating habits in older adults with preserved cognition, whose application takes approximately 25 to 30 min.^15^ However, the cognitive impairment of dementia syndromes generally makes it unfeasible to apply such instruments to older people, which points to the need for screening instruments focused on the caregiver.

The analysis of valid evidence for the internal structure of the RaDID-QC was based on a model with 24 questions on swallowing disorders, addressing behavior, cognition and safety, efficiency, and swallowing skills. These questions were obtained by validating the content and response process. Evidence of the validity of the internal structure is an important step in validating the questionnaire, as it presents the relationship and quantifies the correlation between the questions.^23^^,^^36^^,^^37^ The internal validation results were based on norms that suggest robust and reliable premises from a psychometric standpoint.^23^ Based on the EFA results, the authors reduced the number of questions in RaDID-QC to produce a more concise but still valid and consistent questionnaire, which was achieved by maintaining only questions whose factor loadings were at least 0.45 in magnitude, using varimax orthogonal rotation.

Of all 24 RaDID-QC questions, nine (Q1, Q3, Q6, Q7, Q9, Q18, Q21, Q23, and Q24) were not well correlated with the latent factors (factor loading < 0.45).^33^^,^^38^^,^^39^ These nine questions were removed, and the questionnaire was reduced to a final form with 15 questions (Q2, Q4, Q5, Q8, Q10, Q11, Q12, Q13, Q14, Q15, Q16, Q17, Q19, Q20, and Q22). This decrease did not result in a substantial loss of reliability, since Cronbach's alpha was 0.78 for the complete questionnaire and 0.74 for the final one. The final questionnaire also retained most of the variability of the full questionnaire: the 15 remaining questions explain 71 % of the variance of the full set of 24 questions.

Overall, EFA determined the reduction and defined the dimensionality of the instrument, resulting in a questionnaire that is easier and faster to apply and has greater internal consistency. The reduced questionnaire is also a little redundant since each question had a higher factor loading on just one factor (with the sole exception of Q16, with a high factor loading on factors 3 and 7).

This study has some limitations, such as applying the questionnaire to a population from only one Reference Center. Nevertheless, it is the main geriatric reference service in the city, treating older adults referred by primary health care from all regions of the city. The patients/caregivers were mostly from lower socioeconomic classes, which limited the validity of the application in other populations. Furthermore, only a few formal male caregivers were included, which imposes limitations on assessing the questionnaire for caregivers of the male sex. However, in clinical practice, they represent a minority of caregivers for older adults in most populations. Since the authors included caregivers of all educational levels, the RaDID-QC was administered through interviews. This approach ensured that caregivers who had difficulty reading or completing the questionnaire could understand it more easily. It is important to point out that this study analyzed the characteristics of a screening instrument – therefore, the results should not be interpreted as a clinical diagnosis.

Thus, the RaDID-QC can be considered the first and only dysphagia-related questionnaire to be applied to caregivers of older adults with dementia.

The RaDID-QC is a promising screening tool for dysphagia in older adults with dementia because it is a self-reported questionnaire, is easy to understand, and requires little application time. Additionally, it is internally consistent, reproducible, and valid. It helps to identify early signs and symptoms of OD to avoid swallowing safety and efficiency complications. Therefore, the dissemination of RaDID-QC creates better care management and expands the possibility of preventing worsening and promoting quality of life and health for older adults with dementia. Other validity and reliability parameters will be obtained by applying the questionnaire to larger target populations.

## Conclusion

The RaDID-QC was initially developed with 24 but reduced to 15 questions based on the EFA. It had adequate internal structure and reliability. The original RaDID-QC is a simple, concise, easy-to-administer, fast, and reliable questionnaire.

## Authors’ contributions

Grazielle Duarte de Oliveira, Sayuri Hiasmym Guimarães Pereira dos Santos, Aline Mansueto Mourão and Uriel Moreira Silva were responsible for study conceptualization and design, data collection and analysis, and manuscript writing. Maria Aparecida Camargos Bicalho, Amélia Augusta de Lima Friche, and Laélia Cristina Caseiro Vicente were responsible for study conceptualization and design, supervision in all study´s stages, and manuscript review. All authors read and approved the final version of the manuscript.

## Funding

This work was supported by the Conselho Nacional de Desenvolvimento Científico e Tecnológico (CNPq) – process nº 315133/2021-0; 309953/2018-9; the Pró-Reitoria de Pesquisa of the Universidade Federal de Minas Gerais – Edital PRPq – 09/2019 – Programa Institucional de Auxílio à Pesquisa de Docentes Recém-Contratados; and the Coordenação de Aperfeiçoamento de Pessoal de Nível Superior (CAPES) – process no. 88887.569376/2020-00.

## Declaration of competing interest

The authors declare no conflicts of interest.

## Appendix Supplementary materials

Image, application 1
