# Supplementary material for: Screening for dysphagia in older people with dementia: Evidence of validity based on internal structure and reliability of the Caregiver Questionnaire – RaDID-QC
Source: Clinics (Sao Paulo). 2024 Aug 9;79:100440. doi: 10.1016/j.clinsp.2024.100440 (PMC11369375; doi:10.1016/j.clinsp.2024.100440)

**CLINICS-D-24-00277_Supplementary Material**

**Supplement to “Screening for dysphagia in older people with dementia: Evidence of validity based on internal structure and reliability of the caregiver questionnaire – RaDID-QC”**

**Selection of the number of factors for EFA**

**Table Supplement 1** contains the PCA results for all24 RaDID-QC questions, Figure S1 contains the corresponding correlation matrix, and Figure S2 contains the scree plot of the eigenvalues of the correlation matrix.

**Table Supplement 1** Principal Components Analysis results on the 24 RaDID-QC questions.

| **Eigenvalue decomposition** | | | | | | | | |
| --- | --- | --- | --- | --- | --- | --- | --- | --- |
|  | **Comp. 1** | **Comp. 2** | **Comp. 3** | **Comp. 4** | **Comp. 5** | **Comp. 6** | **Comp. 7** | **Comp. 8** |
| **Eigenval.** | 4.29 | 2.28 | 1.62 | 1.51 | 1.28 | 1.23 | 1.15 | 1.03 |
| **% ex. var.** | 18.00 | 9.57 | 6.80 | 6.35 | 5.35 | 5.16 | 4.81 | 4.31 |
| **% ex. var. (cum)** | 18.00 | 27.57 | 34.37 | 40.71 | 46.06 | 51.22 | 56.04 | 60.35 |
|  | **Comp. 9** | **Comp. 10** | **Comp. 11** | **Comp. 12** | **Comp. 13** | **Comp. 14** | **Comp. 15** | **Comp. 16** |
| **Eigenval.** | 0.97 | 0.91 | 0.87 | 0.83 | 0.79 | 0.67 | 0.67 | 0.62 |
| **% ex. var.** | 4.08 | 3.83 | 3.65 | 3.46 | 3.30 | 2.82 | 2.81 | 2.58 |
| **% ex. var. (cum)** | 64.43 | 68.26 | 71.91 | 75.37 | 78.67 | 81.49 | 84.30 | 86.88 |
|  | **Comp. 17** | **Comp. 18** | **Comp. 19** | **Comp. 20** | **Comp. 21** | **Comp. 22** | **Comp. 23** | **Comp. 24** |
| **Eigenval.** | 0.60 | 0.50 | 0.45 | 0.43 | 0.36 | 0.32 | 0.26 | 0.20 |
| **% ex. var.** | 2.53 | 2.10 | 1.88 | 1.81 | 1.51 | 1.36 | 1.10 | 0.85 |
| **% ex. var. (cum)** | 89.40 | 91.50 | 93.38 | 95.19 | 96.70 | 98.05 | 99.15 | 100.00 |
| **PCA loadings** | | | | | | | | |
|  | **Comp. 1** | **Comp. 2** | **Comp. 3** | **Comp. 4** | **Comp. 5** | **Comp. 6** | **Comp. 7** | **Comp. 8** |
| **Q1** | 0.24 | 0.21 | 0.01 | 0.00 | 0.12 | 0.01 | 0.25 | 0.02 |
| **Q2** | 0.08 | -0.12 | -0.46 | -0.25 | -0.03 | 0.07 | 0.16 | -0.09 |
| **Q3** | 0.18 | 0.14 | -0.09 | -0.28 | 0.18 | -0.33 | -0.07 | -0.13 |
| **Q4** | 0.25 | 0.38 | 0.00 | 0.12 | -0.03 | 0.24 | 0.17 | 0.16 |
| **Q5** | 0.31 | 0.30 | -0.04 | 0.09 | -0.04 | 0.28 | -0.04 | 0.20 |
| **Q6** | 0.09 | 0.09 | 0.26 | 0.33 | 0.03 | -0.04 | 0.33 | -0.12 |
| **Q7** | 0.21 | -0.13 | 0.42 | -0.17 | 0.15 | -0.02 | -0.05 | -0.27 |
| **Q8** | 0.27 | -0.13 | 0.10 | 0.08 | 0.35 | 0.11 | -0.14 | -0.30 |
| **Q9** | 0.16 | -0.27 | -0.04 | 0.31 | 0.12 | 0.26 | 0.07 | 0.02 |
| **Q10** | 0.31 | -0.03 | 0.11 | -0.10 | 0.22 | 0.09 | -0.13 | -0.03 |
| **Q11** | 0.22 | 0.15 | -0.22 | -0.16 | -0.18 | -0.16 | -0.19 | -0.24 |
| **Q12** | 0.27 | 0.26 | -0.23 | -0.01 | -0.19 | 0.10 | -0.01 | 0.01 |
| **Q13** | 0.26 | -0.28 | 0.11 | -0.12 | -0.08 | -0.10 | 0.15 | 0.08 |
| **Q14** | 0.18 | -0.25 | 0.11 | -0.25 | -0.12 | 0.13 | 0.46 | 0.03 |
| **Q15** | 0.13 | -0.29 | -0.22 | -0.11 | -0.11 | 0.45 | -0.05 | -0.04 |
| **Q16** | 0.22 | -0.17 | -0.28 | 0.23 | -0.08 | -0.31 | -0.23 | -0.13 |
| **Q17** | 0.12 | -0.17 | -0.19 | 0.28 | -0.08 | -0.12 | 0.23 | -0.17 |
| **Q18** | 0.27 | 0.15 | 0.21 | 0.05 | -0.03 | -0.15 | -0.10 | -0.18 |
| **Q19** | 0.16 | -0.23 | 0.23 | -0.20 | -0.08 | -0.19 | -0.11 | 0.57 |
| **Q20** | 0.15 | -0.12 | -0.08 | 0.50 | 0.03 | -0.29 | -0.02 | 0.31 |
| **Q21** | 0.09 | -0.28 | 0.11 | 0.10 | -0.49 | 0.04 | -0.12 | -0.15 |
| **Q22** | 0.23 | 0.08 | 0.07 | -0.13 | -0.31 | -0.09 | -0.16 | 0.25 |
| **Q23** | 0.08 | -0.03 | -0.23 | -0.15 | 0.22 | -0.35 | 0.45 | 0.09 |
| **Q24** | 0.08 | -0.16 | -0.21 | -0.01 | 0.48 | 0.14 | -0.28 | 0.28 |
|  | **Comp. 9** | **Comp.10** | **Comp. 11** | **Comp. 12** | **Comp. 13** | **Comp. 14** | **Comp. 15** | **Comp. 16** |
| **Q1** | 0.13 | 0.11 | 0.64 | 0.08 | 0.11 | 0.13 | 0.20 | 0.33 |
| **Q2** | 0.19 | -0.37 | -0.22 | -0.15 | -0.18 | 0.03 | 0.05 | 0.43 |
| **Q3** | -0.30 | -0.09 | 0.28 | -0.38 | -0.08 | -0.23 | -0.23 | 0.08 |
| **Q4** | -0.06 | 0.13 | -0.12 | 0.02 | -0.08 | 0.09 | 0.08 | 0.17 |
| **Q5** | -0.11 | 0.07 | -0.13 | 0.02 | -0.20 | -0.07 | -0.12 | 0.12 |
| **Q6** | 0.38 | -0.26 | -0.16 | -0.30 | 0.21 | -0.42 | 0.21 | -0.04 |
| **Q7** | 0.28 | 0.10 | -0.10 | 0.09 | -0.06 | 0.20 | 0.06 | 0.06 |
| **Q8** | -0.28 | -0.11 | -0.02 | 0.12 | 0.07 | -0.09 | 0.12 | 0.08 |
| **Q9** | -0.24 | -0.36 | 0.09 | 0.10 | 0.26 | -0.10 | -0.45 | 0.00 |
| **Q10** | 0.10 | 0.15 | -0.14 | -0.38 | 0.19 | 0.33 | -0.20 | 0.02 |
| **Q11** | 0.27 | 0.04 | 0.02 | 0.26 | 0.14 | -0.45 | -0.03 | 0.02 |
| **Q12** | 0.11 | 0.11 | -0.11 | 0.09 | 0.08 | -0.04 | -0.26 | -0.34 |
| **Q13** | 0.00 | 0.00 | -0.25 | -0.10 | -0.46 | -0.05 | -0.06 | -0.01 |
| **Q14** | 0.16 | 0.11 | 0.26 | 0.07 | -0.02 | -0.02 | -0.24 | -0.36 |
| **Q15** | -0.06 | -0.14 | 0.22 | 0.01 | -0.09 | 0.00 | 0.46 | -0.17 |
| **Q16** | 0.23 | 0.02 | 0.16 | -0.04 | -0.02 | 0.25 | -0.02 | -0.16 |
| **Q17** | -0.35 | 0.51 | -0.07 | -0.28 | -0.03 | -0.16 | 0.25 | -0.09 |
| **Q18** | -0.21 | -0.20 | -0.08 | 0.38 | -0.26 | 0.01 | 0.19 | -0.18 |
| **Q19** | -0.08 | 0.04 | 0.07 | 0.08 | 0.05 | -0.35 | 0.06 | 0.17 |
| **Q20** | 0.18 | -0.14 | 0.11 | 0.06 | -0.25 | 0.15 | -0.05 | 0.07 |
| **Q21** | -0.04 | 0.22 | -0.09 | 0.11 | 0.28 | 0.04 | -0.10 | 0.48 |
| **Q22** | -0.14 | -0.31 | -0.05 | -0.28 | 0.38 | 0.23 | 0.29 | -0.21 |
| **Q23** | -0.16 | -0.01 | -0.30 | 0.36 | 0.34 | 0.20 | 0.09 | 0.00 |
| **Q24** | 0.25 | 0.25 | -0.14 | 0.05 | 0.13 | -0.20 | 0.15 | -0.08 |
|  | **Comp. 17** | **Comp. 18** | **Comp. 19** | **Comp. 20** | **Comp. 21** | **Comp. 22** | **Comp. 23** | **Comp. 24** |
| **Q1** | 0.05 | 0.08 | 0.10 | 0.23 | 0.00 | 0.01 | 0.34 | 0.11 |
| **Q2** | -0.06 | 0.36 | -0.11 | -0.04 | 0.01 | -0.23 | 0.04 | -0.08 |
| **Q3** | 0.37 | -0.05 | -0.07 | -0.12 | 0.01 | 0.21 | -0.23 | -0.07 |
| **Q4** | -0.13 | -0.03 | 0.31 | -0.22 | 0.04 | 0.18 | -0.30 | -0.54 |
| **Q5** | -0.02 | -0.04 | -0.18 | -0.07 | -0.35 | -0.03 | -0.15 | 0.63 |
| **Q6** | 0.17 | -0.10 | -0.04 | 0.10 | -0.15 | -0.05 | -0.09 | -0.01 |
| **Q7** | -0.10 | 0.27 | -0.37 | -0.19 | -0.05 | 0.48 | -0.02 | 0.00 |
| **Q8** | -0.28 | 0.03 | 0.07 | 0.38 | 0.30 | -0.19 | -0.37 | 0.09 |
| **Q9** | -0.08 | 0.12 | 0.06 | -0.17 | -0.14 | 0.24 | 0.30 | -0.09 |
| **Q10** | -0.04 | -0.39 | -0.08 | -0.12 | 0.01 | -0.41 | 0.26 | -0.12 |
| **Q11** | -0.32 | -0.26 | 0.12 | -0.30 | 0.18 | 0.05 | 0.15 | 0.09 |
| **Q12** | 0.15 | 0.16 | -0.36 | 0.50 | 0.19 | 0.08 | 0.09 | -0.22 |
| **Q13** | 0.02 | -0.21 | 0.42 | 0.32 | 0.08 | 0.28 | 0.27 | 0.10 |
| **Q14** | 0.02 | 0.16 | 0.13 | -0.21 | 0.10 | -0.29 | -0.31 | 0.10 |
| **Q15** | 0.15 | -0.42 | -0.25 | -0.05 | -0.06 | 0.16 | -0.04 | -0.11 |
| **Q16** | -0.14 | 0.05 | 0.20 | 0.15 | -0.54 | 0.00 | -0.25 | -0.09 |
| **Q17** | -0.16 | 0.23 | -0.15 | -0.16 | 0.03 | -0.03 | 0.20 | 0.02 |
| **Q18** | 0.34 | 0.15 | 0.05 | -0.19 | -0.13 | -0.37 | 0.26 | -0.15 |
| **Q19** | -0.23 | 0.03 | -0.25 | 0.12 | -0.22 | -0.16 | -0.02 | -0.28 |
| **Q20** | 0.05 | -0.13 | -0.25 | -0.19 | 0.48 | -0.01 | -0.11 | 0.07 |
| **Q21** | 0.44 | -0.03 | 0.07 | 0.04 | 0.07 | -0.03 | -0.16 | 0.02 |
| **Q22** | -0.07 | 0.24 | 0.17 | -0.08 | 0.20 | 0.13 | 0.01 | 0.22 |
| **Q23** | 0.09 | -0.25 | -0.10 | 0.01 | -0.11 | 0.10 | -0.08 | 0.09 |
| **Q24** | 0.37 | 0.25 | 0.25 | -0.10 | 0.06 | 0.04 | 0.01 | 0.06 |

Caption: Q1 to Q24, Questions 1 through 24, Comp. 1 to Comp. 24, Principal components 1 through 24, Eigenval, Eigenvalues, % ex.var., Percentage of explained variance, % ex. var. (cum), Cumulative percentage of explained variance, PCA, Principal Components Analysis.

**Figure Supplement 1** Correlation matrix between all 24 RaDID-QC questions. Caption: Q1 to Q24, Questions 1 through 24.


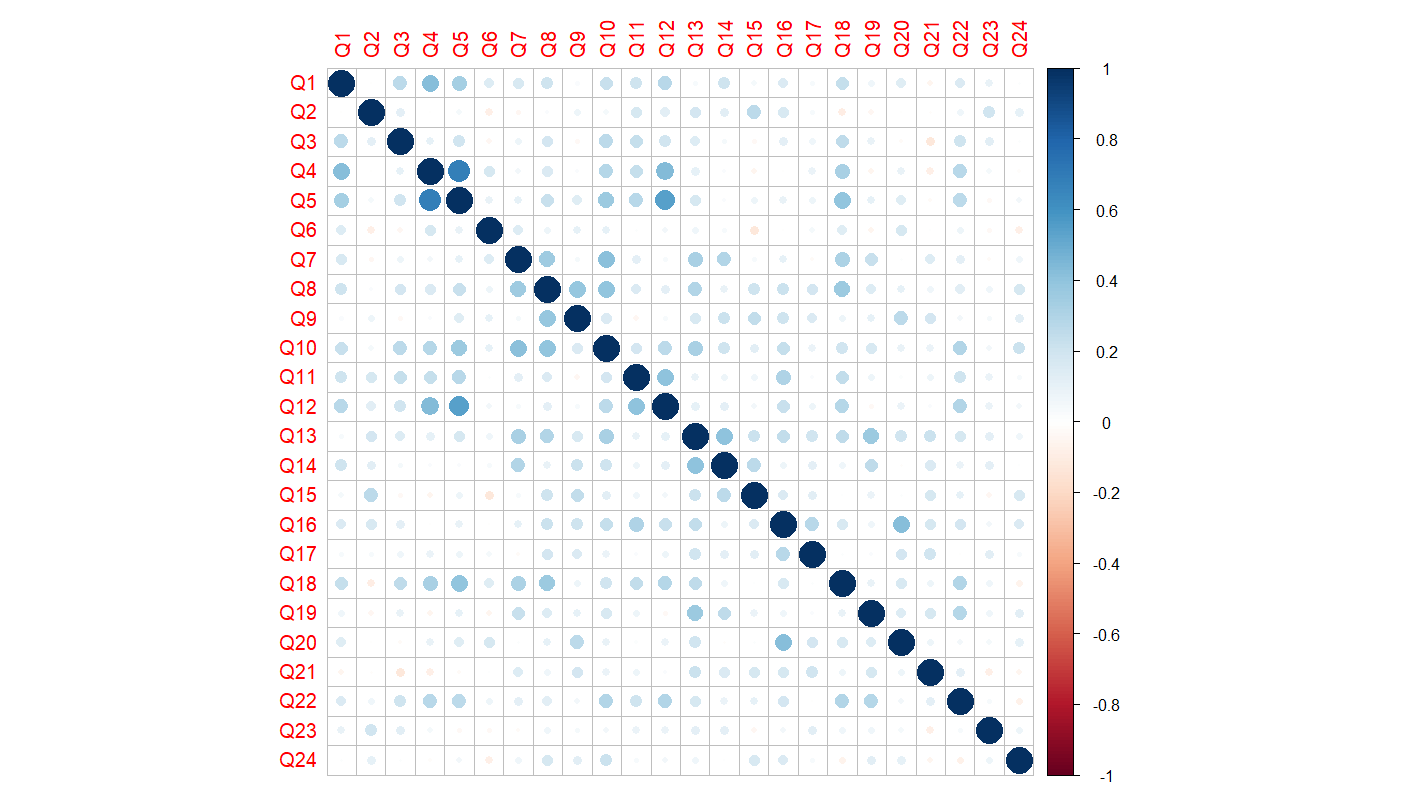


**Figure Supplement 2** **Scree plot of the eigenvalues of the correlation matrix of the 24 RaDID-QC questions.** Caption: Comp. 1 to Comp. 10, Principal components 1 through 10.


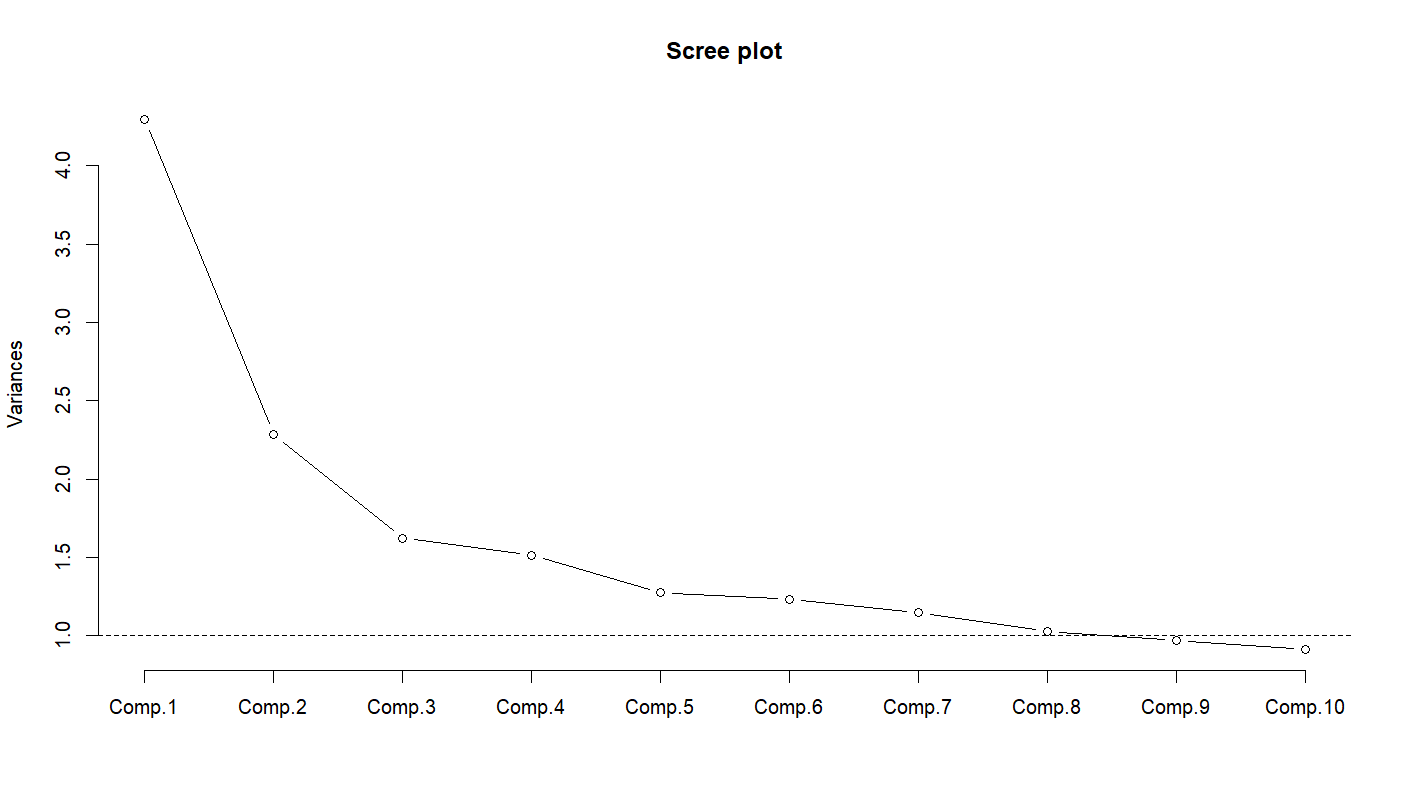

Supplement: Supplementary file 1 [file mmc1.docx]
